# Jarisch-Herxheimer Reaction After Cephalosporin Administration in Syphilis

**DOI:** 10.7759/cureus.12750

**Published:** 2021-01-17

**Authors:** Caitlin McKenzie, Jennifer Olges

**Affiliations:** 1 Internal Medicine-Pediatrics, University of Louisville, Louisville, USA; 2 Internal Medicine, University of Louisville, Louisville, USA

**Keywords:** jarisch-herxheimer, syphilis, cephalosporin, hypotension

## Abstract

The Jarisch-Herxheimer reaction (JHR) is a well-described entity most commonly occurring after the treatment of syphilis with penicillin. Patients often experience flu-like symptoms, in addition to worsening of cutaneous manifestations of syphilis. Severe reactions are uncommon but may include signs of exaggerated systemic inflammatory response. We report a case of a 33-year-old male with secondary syphilis who was treated with ceftriaxone and subsequently developed fluid-refractory hypotension requiring vasopressor administration and intensive care unit admission. To our knowledge, this is the first report of severe hypotension as a result of JHR in a patient with syphilis who was treated with cephalosporin antibiotics.

## Introduction

The Jarisch-Herxheimer reaction (JHR) is a well-reported phenomenon most commonly occurring after the treatment of syphilis with penicillin. Patients often experience flu-like symptoms, which include but are not limited to fevers, chills, and myalgias, in addition to worsening of skin rash and other cutaneous manifestations of the disease. The reaction typically occurs within the first four to 12 hours after antibiotic administration and self-resolves within 24 hours. The mechanism of the JHR remains unknown, but cytokine release in response to treponemal lipoproteins is thought to be implicated in its pathogenesis. Severe reactions are uncommon but may include signs of an exaggerated systemic inflammatory response, including hemodynamic instability and thermoregulatory dysfunction. Herein, we report a case of secondary syphilis treated with ceftriaxone who subsequently developed fluid-refractory hypotension requiring vasopressor administration. This article was previously presented as a poster at the 2018 Kentucky American College of Physicians meeting on October 3, 2018.

## Case presentation

A 33-year-old male with human immunodeficiency virus (HIV), not on highly active antiretroviral therapy (HAART) for the last two years, presented to an outside emergency department with a three-week history of subjective fever, arthralgias, myalgias, and a diffuse painful papular upper body rash. The rash had progressed from his trunk to involve his face, arms, and upper legs. He reported painless genital lesions preceding the diffuse rash, as well as two unprotected sexual encounters approximately one month prior. The patient took no home medications and had no known medication allergies. In the emergency department, he was found to have a temperature of 100.9°F, a heart rate of 130 beats per minute, and a respiratory rate of 22 breaths per minute. Urinalysis was not suggestive of infection, and chest radiograph demonstrated no infiltrate. Blood and urine cultures were obtained. He was given 1 L bolus of intravenous fluids and 1 g of ceftriaxone intravenously for presumed sepsis. He was subsequently transferred to our institution for further workup and management.

**Figure 1:**
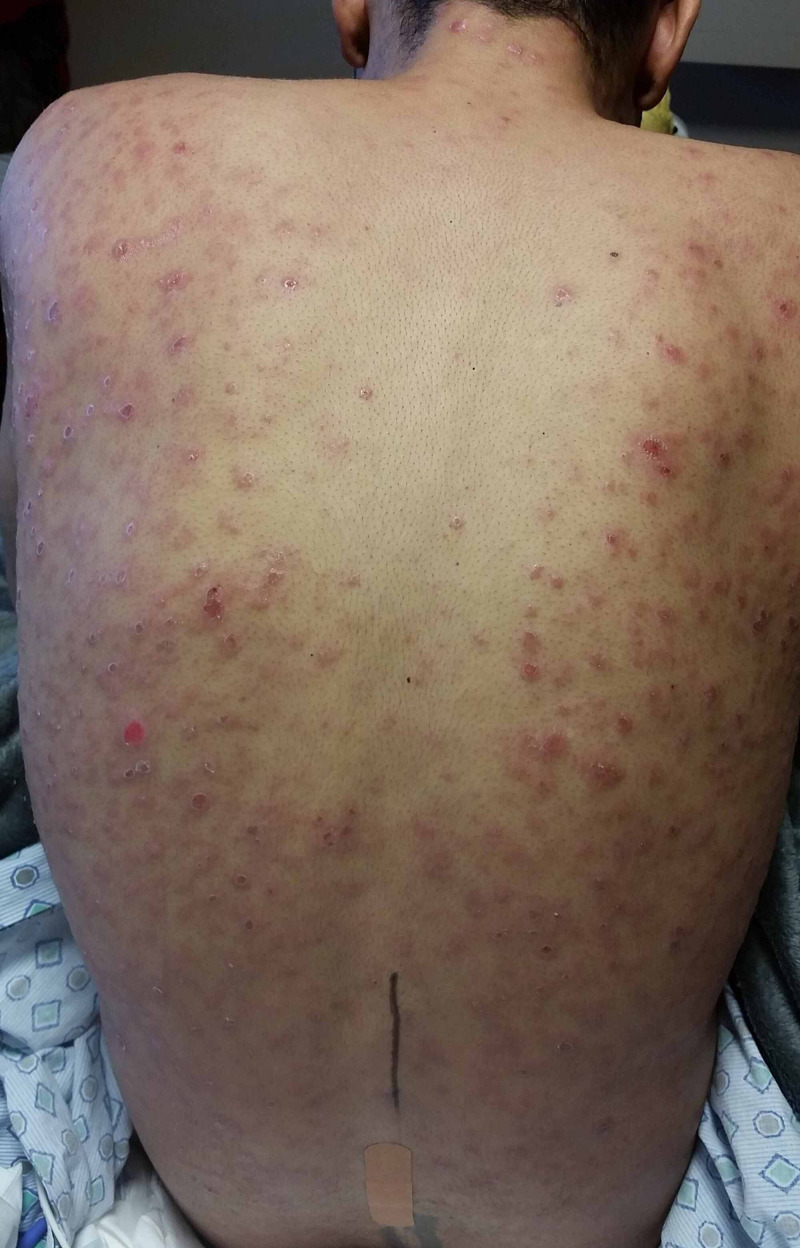
Scaly, erythematous papular lesions located diffusely over the patient's back.

**Figure 2:**
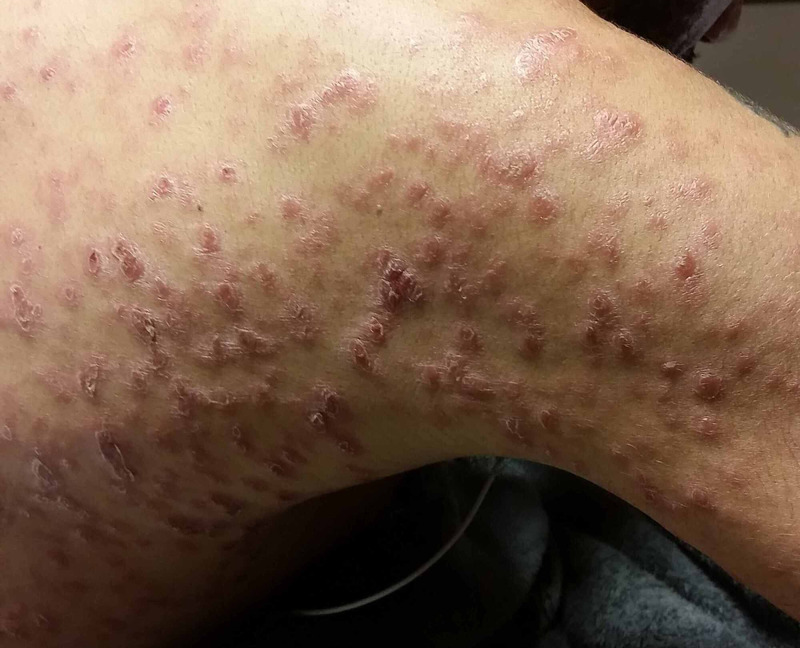
A closer view of the papulosquamous lesions over the patient's posterolateral arm and upper back.

Upon arrival to our emergency department six hours later, the patient was febrile to 102.8°F with a heart rate of 161 beats per minute and systolic blood pressure initially 89 mmHg. Physical exam was remarkable for normal mentation, tachycardia but no murmurs, and clear lung fields bilaterally. There were erythematous scaly papular and plaque-like lesions over the face, back, chest, arms, abdomen, and upper legs (Figures 1, 2). The rash spared the palms and soles. He also had an erythematous papule on the mucosa of the lower lip. The patient remained hypotensive, with a blood pressure of 82/53 mmHg, despite 3 L of normal saline. A central venous catheter was placed, and a norepinephrine drip was started. The patient also received empiric antibiotic treatment with vancomycin and cefepime for presumed septic shock. The patient was admitted to the medical intensive care unit, where he required less than 24 hours of vasopressor administration with norepinephrine to maintain adequate blood pressure.

Blood and urine cultures obtained at the outside hospital prior to the first antibiotic dose were negative for bacterial growth. Broad-spectrum antibiotics were discontinued once cultures showed no growth for over 48 hours and the patient remained clinically stable. Blood cultures, fungal cultures, acid-fast bacilli smear and cultures, cerebrospinal fluid (CSF) cultures, cryptococcal serum and CSF antigens, Histoplasma galactomannan urine antigen, Neisseria gonorrhea polymerase chain reaction (PCR), and Chlamydia trachomatis PCR obtained at our facility were all negative. CSF studies demonstrated no red blood cells, no white blood cells, glucose 53 mg/dL (reference range 40-70 mg/dL), and protein 49.5 mg/dL (reference range 10-45 mg/dL), which was not thought to be consistent with aseptic meningitis. Rapid plasma reagin (RPR) was positive, and syphilis serum titer was 1:256 dL. CSF RPR was negative, and CSF Treponema pallidum IgG by immunofluorescence assay was nonreactive. The patient was also noted to have a CD4 T-cell count of 162/mm^3^ (reference range 338 to 1448 per mm^3^) and HIV-1 ribonucleic acid (RNA) of 456,000 copies/mL. Dermatology was consulted and deferred skin biopsy as exam findings were clinically consistent with secondary syphilis. The patient was treated with 2.4 million units of intramuscular benzathine penicillin. The remainder of his hospital course was uncomplicated, and he was discharged home after three days with close follow-up with Infectious Diseases with plans to resume HAART as an outpatient.

## Discussion

Since 2001, the incidence of primary and secondary syphilis in the United States has increased dramatically. In 2018, the rate of primary and secondary syphilis was 10.8 cases per 100,000 population, which marked a 14.9% increase from 2017 and a 71.4% increase from 2014 [1]. Syphilis is caused by the spirochete Treponema pallidum*,* and the infection’s manifestations depend upon the stage of the disease. Primary syphilis classically presents as a non-tender genital chancre. The chancre may go unnoticed if not in a highly visible location, and thus treatment often is delayed until further progression of the disease. Secondary syphilis typically occurs two to eight weeks after the initial infection, with a characteristic rash, often involving the trunk, face, and extremities. Classically, the lesions may involve the palms and soles, although lack of involvement does exclude the diagnosis. Moist, heaped-up intertriginous lesions known as condyloma lata may be present. Skin lesions are highly infectious, and biopsy of these lesions examined under dark-field microscopy will reveal treponemes. In addition to classic dermatologic manifestations, secondary syphilis may also present with aseptic meningitis, patchy alopecia, and other mucocutaneous involvement. If untreated, the infection may enter a latent phase in which there are no signs or symptoms of the disease, but serological tests remain positive. Endarteritis is the characteristic presentation of tertiary syphilis and may manifest with neurologic involvement, cardiovascular involvement, or gummatous syphilis. Neurologic sequelae include focal ischemia due to meningovascular involvement and general paresis. Tabes dorsalis, for instance, is defined by syphilitic involvement of the posterior columns of the spinal cord, which leads to sensory ataxia of the lower extremities. Cardiovascular involvement may lead to aortitis and aortic aneurysm. Gummatous disease presents as destructive lesions of the skin, soft tissue, and bony structures. Syphilis may also be transmitted from mother to fetus, and congenital syphilis has varied manifestations but will not be discussed in detail in this report.

The diagnosis of syphilis often begins with nontreponemal screening tests, including RPR and venereal disease research laboratory (VDRL). Patients with positive screening tests should be tested for specific treponemal markers, such as fluorescent treponemal absorption assay, treponemal particle agglutination, enzyme immunoassays, and chemiluminescence immunoassays. CSF analysis should be performed if there are any neurologic or ophthalmic symptoms at any stage of the disease.

The treatment of choice for all stages of syphilis is penicillin G. Primary, secondary, and early latent disease may be treated with a single injection of 2.4 million units of penicillin G benzathine. Tertiary syphilis and late latent disease require treatment duration of three weeks with daily benzathine penicillin injections. Neurosyphilis should be treated with 3 to 4 million units of intravenous crystalline benzathine penicillin every four hours for three weeks [2].

One of the most common complications of treatment is the Jarisch-Herxheimer reaction (JHR). Classically described with penicillin administration in patients with syphilis, the JHR is also known to occur in other spirochetal diseases, including leptospirosis and Borrelia infection. One prospective observational study reported a higher incidence of JHR in patients with HIV who were treated for syphilis compared to non-HIV infected patients (34.6% vs. 25.2%, respectively), although this difference was not statistically significant [3]. Symptoms of JHR include fevers, chills, headache, myalgia, and worsening of skin manifestations. The reaction typically occurs within the first four to six hours after induction of therapy, with peak symptoms occurring around six to eight hours and resolution of symptoms by 16 to 24 hours [4].

The exact mechanism of JHR remains unclear. The most widely accepted theory is that lipopolysaccharides, a constituent of bacterial cell membranes, are released during exposure to certain antibiotics and may cause a systemic inflammatory response [4,5]. It is postulated that treponemal lipoproteins, once released, undergo phagocytosis by macrophages, which then secrete tumor necrosis factor alpha. Other cytokines, including interleukins IL-6 and IL-8, are also implicated in the inflammatory response to these lipoproteins. In the early phases of the reaction, patients may exhibit vasoconstriction and elevated blood pressure. Later, there is often vasodilation and decreased peripheral resistance, leading to hypotension. There have been few reports of severe reactions requiring vasopressors for hypotension in patients with JHR after treatment of leptospirosis [6,7]. However, most patients recover spontaneously and require minimal supportive care.

The patient described here had a profound reaction to cephalosporin administration with fluid-refractory hypotension but had subsequent clinical improvement and no further complications during the remainder of his hospital stay. A comprehensive evaluation for other infectious etiologies of his symptoms was unrevealing. He had no known allergies and tolerated further cephalosporin administration; thus, anaphylaxis was not likely implicated as the etiology of his symptoms. Given the timing of his symptoms in relation to antibiotic administration and diagnosis of secondary syphilis, his clinical presentation was thought to be most consistent with JHR.

## Conclusions

We report a case of a patient who required admission to the intensive care unit and vasopressor support in the setting of presumed JHR. To our knowledge, this is the first report of a patient with syphilis who experienced severe hypotension from presumed JHR requiring vasopressor administration after administration of a cephalosporin antibiotic. Providers must be aware that JHR may occur in patients who are treated for syphilis, and, although rare, severe reactions may occur.
